# How to Derive Biological Information from the Value of the Normalization Constant in Allometric Equations

**DOI:** 10.1371/journal.pone.0001932

**Published:** 2008-04-09

**Authors:** Pekka Kaitaniemi

**Affiliations:** Hyytiälä Forestry Field Station, University of Helsinki, Korkeakoski, Finland; University of Arizona, United States of America

## Abstract

Allometric equations are widely used in many branches of biological science. The potential information content of the normalization constant *b* in allometric equations of the form *Y = bX^a^* has, however, remained largely neglected. To demonstrate the potential for utilizing this information, I generated a large number of artificial datasets that resembled those that are frequently encountered in biological studies, i.e., relatively small samples including measurement error or uncontrolled variation. The value of *X* was allowed to vary randomly within the limits describing different data ranges, and *a* was set to a fixed theoretical value. The constant *b* was set to a range of values describing the effect of a continuous environmental variable. In addition, a normally distributed random error was added to the values of both *X* and *Y*. Two different approaches were then used to model the data. The traditional approach estimated both *a* and *b* using a regression model, whereas an alternative approach set the exponent *a* at its theoretical value and only estimated the value of *b*. Both approaches produced virtually the same model fit with less than 0.3% difference in the coefficient of determination. Only the alternative approach was able to precisely reproduce the effect of the environmental variable, which was largely lost among noise variation when using the traditional approach. The results show how the value of *b* can be used as a source of valuable biological information if an appropriate regression model is selected.

## Introduction

Allometric scaling laws, which can be used to characterize many biological systems [1]–[3], take the form *Y = bX* ^α^, which represents the dependence of the variable *Y* on the variable *X* as a function that involves the normalization constant *b* and the scaling exponent *a*. This exponent has been the focus of theoretical and empirical studies because it often seems to have a constant value specific to a particular biological relationship.

In the process of exploring *a*, only a few studies have directly considered the potential information content of the normalization constant *b* [3]–[6], and unexplored potential for using the normalization constant *b* to depict biologically important information remains. An example can be drawn from Mäkelä and Valentine [7], who used empirical data to demonstrate how crown ratio (*r_c_*) influences the scaling relationship in trees, suggesting that the scaling between foliage mass (*M_F_*) and woody mass (*M_T_*) can be approximated by the relation *M_F_* ∝ *(r_c_^2^M_T_)^z/(az+1)^*, where z is the fractal dimension of foliage and *a* is defined through the relationship *r*_k+1_ = *n* ^−*a*/2^ *r*_k_, where *n* is the number of daughter branches and *r_k_* is the diameter of branches at the *k*'th branching level. In their recent paper [6], Enquist et al. also suggest a number of direct biological interpretations for the value of *b*.

In traditional approaches, the values of both *a* and *b* are estimated by one of the several regression methods available [8]. In the process of parameter estimation, however, the values of the two parameters, *a* and *b*, are essentially mathematically dependent on each other [9]. If a fixed value is used for *a* instead of an empirical one, the estimation algorithm will change the value of *b* so that the amount of residual variation remains as small as possible. I will show how this property of allometric equations can be used to derive biological information from the value of *b* by assuming a fixed theoretical exponent *a*, and how this information may be lost if both *a* and *b* are empirically determined by regression.

I have deliberately chosen to consider the scaling laws as models for biological phenomena [10]. A model can be considered a useful representation of a biological system [11], if it 1) is useful for system management, 2) provides insight, 3) gives accurate predictions, 4) is simple and elegant, 5) bears generality, 6) is insensitive to assumptions and 7) has a low construction cost. Models based on simulated datasets will demonstrate that there is great potential for using the normalization constant *b* as a simple measure for characterizing environmental variability that seems typical of comparisons involving the allometric scaling relationships observed in different sets of biological data [12]–[15].

## Results and Discussion

In the present analysis, I ignored the details of the relation used by Mäkelä and Valentine [7] and assumed a system that has a constant scaling exponent 0.75 and a biological phenomenon comparable with crown ratio affecting the scaling relation. This can be represented by the formula *M_F_ = b(r_c_^2^M_T_)^0.75^*, which can be rewritten as *M_F_ = br_c_^1.5^M_T_^0.75^*. The effect of *r_c_* can now be incorporated into a single normalization constant *b_adj_ = br_c_^1.5^*, where it is assumed that an otherwise constant biological relationship is modified by an environmental variable *r_c_* that is included in the value of the normalization constant. This enables the use of the empirically detected values of *b* for characterizing the effect of the environment on the system, i.e., detecting the actual value of *b_adj_*.

**Figure 1:**
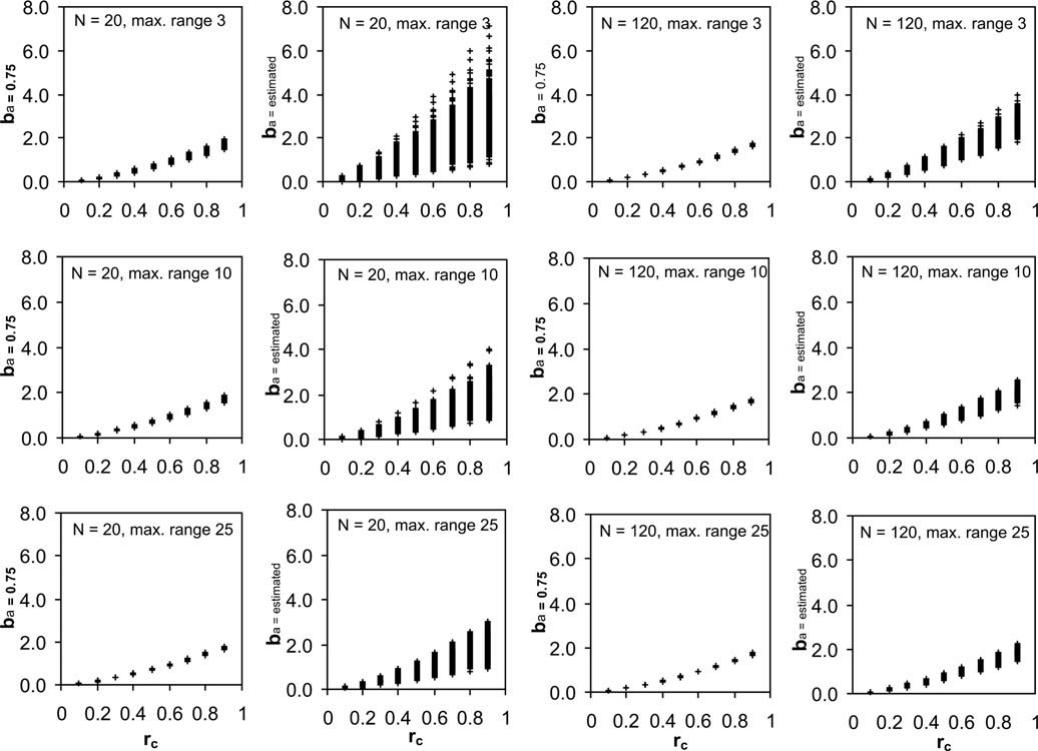
The effect of sample size and data range on the values of the normalization constant. The normalization constant *b* was estimated by nonlinear regression for sets of allometric data generated with the function *Y = 2r_c_^1.5^X^0.75^* , where *2r_c_^1.5^* = *b* and where random variation was added to both *Y* and *X*. Nonlinear regression was then used to estimate the value of *b* from the data generated either by assuming a constant exponent *a* = 0.75 (*b*_a = 0.75_) or by allowing the value of *a* to be estimated by regression (producing *b*_a = estimated_). For each value of *r_c_*, the graphs show 600 instances of normalization constants estimated using data with either N = 20 or N = 120, and with a range approaching 3-fold, 10-fold or 25-fold differences in the values of *X*. Each instance is shown as a ‘+’ sign.

When both *a* and *b* were allowed to vary during the estimation of the parameter values, nonlinear regression systematically underestimated the true scaling exponent (*0.75*), especially in small datasets where the data range was narrow (i.e., the relationship between *Y* and *X* was often weak) (Table 1). When the scaling exponent *a* was set to the fixed value of 0.75, the effect of *r_c_* on normalization constant was portrayed clearly even with a narrow data range, as can be seen in the values of *b*_a = 0.75_, which now closely match the actual value of *b_adj_* (Fig. 1). In contrast, a large amount of noise remained in the value of *b* (shown as *b*_a = estimated_ in Fig. 1), where both *a* and *b* were determined by regression. Both analysis methods produced an almost equal coefficient of determination (*r^2^*) with slight but biologically unimportant differences detectable only at the third decimal place (Table 1). Instead, in the regressions with fixed *a*, the variability that would otherwise have characterized the value of *a* (Table 1) became included in the value of *b*_a = 0.75_ in a way that allowed the capture of additional biological information from the data (Fig. 1).

**Table 1:** The fit of allometric models with either a fixed (subscript 0.75) or an empirical scaling exponent (subscript *a*).

Approximate data range	*r*_0.75_^2^	*r*_0.75_	(SD)	*r_a_* ^2^	*r_a_*	(SD)	*a*	(SD)
a) N = 20								
2	0.488	0.698	(0.116)	0.487	0.698	(0.117)	0.562	(0.142)
3	0.676	0.822	(0.068)	0.677	0.823	(0.067)	0.642	(0.110)
4	0.757	0.870	(0.056)	0.759	0.871	(0.055)	0.672	(0.095)
5	0.808	0.899	(0.042)	0.810	0.900	(0.049)	0.692	(0.087)
9	0.897	0.947	(0.025)	0.898	0.947	(0.025)	0.718	(0.069)
24	0.968	0.979	(0.009)	0.968	0.979	(0.009)	0.739	(0.042)
b) N = 120								
2	0.488	0.699	(0.044)	0.489	0.699	(0.044)	0.554	(0.054)
3	0.677	0.823	(0.027)	0.678	0.824	(0.027)	0.635	(0.042)
4	0.760	0.872	(0.019)	0.761	0.872	(0.019)	0.668	(0.036)
5	0.810	0.900	(0.016)	0.810	0.900	(0.016)	0.687	(0.035)
9	0.896	0.946	(0.009)	0.896	0.947	(0.009)	0.715	(0.025)
24	0.958	0.979	(0.004)	0.958	0.979	(0.002)	0.743	(0.017)

Both the coefficient of determination (*r^2^*) and the correlation coefficient (*r* with its standard deviation SD) between the actual and model-predicted values are shown. Column *a* shows the average scaling exponent when it was empirically estimated. Each cell in the table shows the mean values for 600 simulated datasets where the number of observations (N) was either 20 or 120. SD indicates the amount of variability in the model fit obtained (columns after *r*) or in the estimate of *a* (column after *a*).

Taken altogether, the use of a fixed *a* enabled an efficient way of detecting and modeling the true value of normalization constant (i.e., *b_adj_*, or *b*_a = 0.75_ in Fig. 1). The resulting model clearly meets the quality criteria listed by Haefner [11]: it is useful for system management, because it captures the value of the parameter that actually controls the scaling relationship; it provides insight, because it enables the detection and modeling of the actual biological process, which would otherwise have remained largely camouflaged by noise variation; it is at least as simple and accurate as the traditional approach; it has great potential for generality, because it reduces the number of unknown parameters in the equation from two to one and provides a robust basis for the biological interpretation of the remaining one; it also has as low a construction cost as the traditional approach. What remains arguable is the sensitivity of the approach to the assumption of a constant scaling exponent *a*, because there may be no consensus about its true value [12]–[15]. If *a* varies instead of *b*, then the approach will fail to produce informative results. However, even this will be only a matter of testing the model fit with different theoretically predicted values of *a*.

These findings have two important biological consequences. First, they show how the value of the normalization constant may be an important source of biological information. It is possible to foresee a whole set of biological models rewritten to include the effect of environmental factors in the value of the normalization constant. Currently all this information may be lost among the noise variation that characterized the values of the allometric parameters obtained using the traditional approach. Second, the findings also demonstrate that the value of the scaling exponent may be misleading if it is allowed to vary during the parameter estimation, which is a result that also holds true with the common linear regression methods [10]. This implies that it may be informative to test the fit of the scaling models by using a set of fixed exponents before rejecting any of its theoretical values [16]. It should be noted, however, that modeling the allometric relationships operates with different criteria than examining the scaling relationships statistically. Statistical tests require that the data meet several specific assumptions that should be checked before making any inference about the values of parameters.

## Materials and Methods

A set of artificial datasets were generated using the SAS statistical software package (SAS Institute Inc., Cary, NC, USA). The sets resembled those that are often encountered in biological studies, i.e., relatively small samples, which include measurement error or uncontrolled environmental variability. Two sample sizes of *X*, small and moderate, were repeatedly generated such that there were 600 instances with N = 20 and 600 instances with N = 120. The values of *X* in the allometric equation with the form *Y = bX* ^α^ were generated using the function *ranuni*, which produces any value within a given range with equal probability, hence assuming a uniform distribution. The data generated represented six categories of range that the variable *X* might have in organisms, starting from the minimum value *X* = 5 and ranging maximally from 3- to 25-fold differences (i.e. between 5 and 15 and 5 to 125). The actual range of *X* within each category was variable because of the use of uniformly distributed random values for generating *X*, and the addition of normally distributed error (see below), but in more than 50% of the individual datasets the true range within each category was at least *5×(range multiplier − 1)*, where the *range multiplier* was 3 for 3-fold differences, 4 for 4-fold differences, etc. This method was sufficient for incorporating the effect of data range in the analysis.

Values of *Y* for each *X* were first calculated with the equation *Y = 2X^0.75^*, i.e., using *b* = 2 and *a* = 0.75. After the calculation of *Y* with the allometric equation, the normally distributed random error was added to each value of both *X* and *Y* using the SAS function *rannor*, and assuming that the error variance equaled 25% of the value of both *X* and *Y*. The addition of the random error was done as the last step after calculating the actual values of X and Y to avoid unnecessary error propagation. The effect of a range of values of *r_c_* (0.1 to 0.9 with 0.1 steps) was added to the artificial datasets by multiplying the values of *Y* with *r_c_^1.5^* to achieve *b_adj_* = 2 *r_c_^1.5^*.

Nonlinear allometric models were fitted to the data generated in two different ways using the SAS procedure NLIN: either both *a* and *b* were determined by the procedure, or only *b* was determined and *a* was directly set to its “theoretical” value of 0.75. The corresponding differences in the values of *b* and model fit were examined. The coefficient of determination *r^2^* was used as the measure of model fit. In each dataset, its calculation was based on the correlation coefficient between the original values and the values of *Y* calculated with the model-estimated parameters *a* and *b*.

## References

[1] Savage VM, Gillooly JF, Woodruff WH, West GB, Allen AP (2004). The predominance of quarter-power scaling in biology.. Funct Ecol.

[2] Marquet PA, Quinones RA, Abades S, Labra F, Tognelli M (2005). Scaling and power-laws in ecological systems.. J Exp Biol.

[3] West GB, Brown JH (2005). The origin of allometric scaling laws in biology from genomes to ecosystems: towards a quantitative unifying theory of biological structure and organization.. J Exp Biol.

[4] Tang H, Mayersohn M (2005). A mathematical description of the functionality of correction factors used in allometry for predicting human drug clearance.. Drug Metab Dispos.

[5] Etienne RS, Apol MEF, Olff H (2006). Demystifying the West, Brown & Enquist model of the allometry of metabolism.. Funct Ecol.

[6] Enquist BJ, Kerkhoff AJ, Stark SC, Swenson NG, McCarthy MC (2007). A general integrative model for scaling plant growth, carbon flux, and functional trait spectra.. Nature.

[7] Mäkelä A, Valentine H (2006). Crown ratio influences allometric scaling in trees.. Ecology.

[8] Isobe T, Feigelson ED, Akritas MG, Babu GJ (1990). Linear regression in astronomy.. I. Astrophys J.

[9] Lumer H (1936). The relation between *b* and *k* in systems of relative growth function of the form *y = bx^k^*.. Am Nat.

[10] Kaitaniemi P (2004). Testing the allometric scaling laws.. J Theor Biol.

[11] Haefner JW (1996). Modeling biological systems.

[12] Dodds PS, Rothman DH, Weitz JS (2001). Re-examination of the “3/4-law” of metabolism.. J Theor Biol.

[13] Chen XW, Li BL (2003). Testing the allometric scaling relationships with seedlings of two tree species.. Acta Oecol.

[14] White CR, Seymour RS (2005). Sample size and mass range effects on the allometric exponent of basal metabolic rate.. Comp Biochem Physiol A.

[15] Muller-Landau HC, Condit RS, Chave J, Thomas SC, Bohlman SA (2006). Testing metabolic ecology theory for allometric scaling of tree size, growth and mortality in tropical forests.. Ecol Lett.

[16] Mahmood I (2006). Prediction of drug clearance in children from adults: a comparison of several allometric methods.. Br J Clin Pharmacol.

